# Medical simulation-based education improves medicos' clinical skills

**DOI:** 10.7555/JBR.27.20120131

**Published:** 2012-12-24

**Authors:** Zhaoming Wang, Qiaoyu Liu, Hai Wang

**Affiliations:** a Department of Surgery, The First School of Clinical Medicine, Nanjing Medical University, Nanjing, Jiangsu 210000, China;; b Department of General Surgery, Anhui Province Hospital, Hefei, Anhui 230001, China

**Keywords:** medical simulation-based education, medicos, clinical skills

## Abstract

Clinical skill is an essential part of clinical medicine and plays quite an important role in bridging medicos and physicians. Due to the realities in China, traditional medical education is facing many challenges. There are few opportunities for students to practice their clinical skills and their dexterities are generally at a low level. Medical simulation-based education is a new teaching modality and helps to improve medicos' clinical skills to a large degree. Medical simulation-based education has many significant advantages and will be further developed and applied.

## INTRODUCTION

Clinical medicine is a practical subject and clinical skill training is a very important part of clinical teaching and plays a key role in the training of medical students and residents towards a qualified clinician. In recent years, traditional clinical teaching is facing more and more challenges in China due to increasing enrollment of medical students and patients' increased awareness of self protection. The mismatch between traditional clinical teaching, which mainly uses patients for students' skill training, and clinical work is becoming more and more apparent. As a result, the current trend is to use simulation as a teaching tool for training and evaluating their students[1].

## CHALLENGES IN CLINICAL SKILLS TRAINING

To adapt to modern medical development and to cultivate modern medical talents, the Institute for International Medical Education has published the global minimum essential requirements in medical education (GMER). GMER contains 60 criteria and includes 7 major parts: professional values, attitudes, behavior and ethics, scientific foundation of medicine, communication skills, clinical skills, population health and health systems, management of information, critical thinking and research[2]. GMER fully reflects the trend of modern medical education revolution and development. Nowadays, a physician must have comprehensive qualities such as good medical ethics, solid medical knowledge, practiced clinical skills and so on. Among these abilities, clinical skills play a key role in the cultivation of medical students. In the United States and Europe, “See one, do one, and teach one” is the principle of clinical practice, and medical simulation education is the location between “see one” and “do one”[3] by bridging the gap between students and physicians. However, the training of clinical skills has always been a difficult problem in medical education. With the development of science and technology, bringing modern technology to medical education makes significant sense and helps medical education and medicos' cultivation.

Health professional education needs to be redesigned to equip students with the knowledge, skills, and attitudes which they need to function safely and effectively in health care delivery in the 21^st^ century[4]. In China, educators pay more attention to theoretical study than to skill training. Teachers play the initiative role in our traditional medical education mode and students are mainly evaluated by their scores. There is no specific course for skill training, which is just merged into other courses. Thus, students acquire a large amount of theoretical knowledge but they do not have enough opportunities to learn and practice their clinical skills. As a result, students lack qualified practical abilities, creating a significant gap between medical theoretical knowledge and clinical skills. However, theory is just the basis of clinical medicine whose essence is to treat and cure patients. It is impossible to be a good physician without well-rounded skills. Stressing clinical skills is the key to both medical education and educational reform, and it is now a matter of great urgency.

The expansion of enrollment at medical schools in China has led to the situation that there are too many medicos in affiliated hospitals. Though medical schools are working hard to improve the teaching mode and establish more reasonable allocation of teaching resources, clinical teaching does not get better in essence. The number of teachers and patients is limited. Students do not have enough resources and thus they lack ample manual opportunities. Moreover, if a large number of inadequately trained medicos pour into wards, not only can the teaching objective not be reached, but it also jeopardizes the clinical work of teaching hospitals. Medicolegal conflict is a serious issue of the society today. Teaching hospitals and physicians will not allow medicos to do the operation themselves in order to reduce medical malpractice risks. It reduces the chance for students' skill training further. Students can only learn theoretical knowledge and do not have a good platform to practice their clinical skills.

With the development of modern society, the requirements for medical service become exigent. More and more attention is paid to patients and medical ethics; patients' self-protection awareness is enhanced. In hospitals, many patients do not interact with medicos for physical examinations and they will not allow students to perform medical procedures on them. The new Regulation on the Handling of Medical Accidents and Law on Practicing Physicians protects patients' interest by law while failing to recognize interns' legal qualifications. It makes the scarce resources less available and students cannot train their clinical skills by practicing on patients. The traditional teaching method is facing a great challenge. Nevertheless, simulated training can provide both medical students and patients a safe environment for practice and errors[5].

## APPLICATION OF SIMULATION EDUCATION TO IMPROVE CLINICAL SKILLS

Our school has established the medical simulation center to provide students a platform to train their clinical skills. They can practice various kinds of manipulating procedures repeatedly to become proficient in them. Issenberg identified that the most important factor in separating the elite performer from others is the amount of “deliberate practice” that an individual obtains[6]. Repetition of practice can help medical students achieve competency and confidence to perform the manual work well. Besides, through simulation training, they can have a deeper understanding of what they have learned from books. It helps to form medicos' clinical thinking abilities.

**Fig 1:**
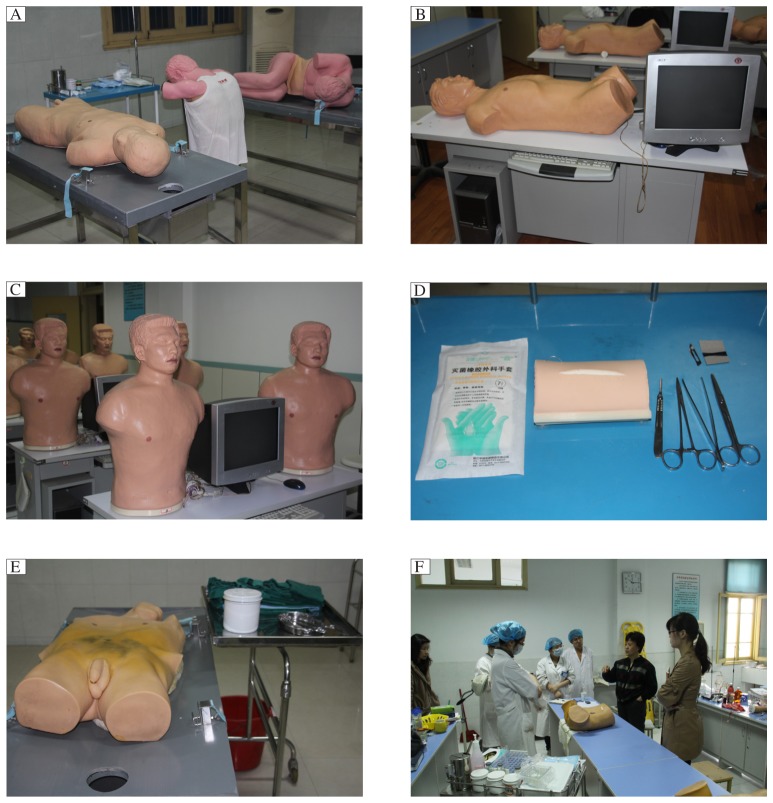
Simulation models in clinical skills training. A: Multiple function puncture model, thoracentesis model, and lumbar puncture model. B and C: Standardized patients for physical examination, heart and lung auscultation. D and E: Models for incision, suturing and asepsis. F: Simulation training in gynecologial examination.

Due to the training objective and the need of teaching, we have introduced many medical simulation models in our teaching programs. In diagnostics, we have obtained machines for complete blood count and urinalysis, microscopes, equipment for staining and so on. In internal medicine, we have obtained models for peritoneal puncture, bone marrow aspiration, thoracentesis, and lumbar puncture (***Fig. 1A***). Students can practice paracentesis on these models for as many times as they want. In addition, electronic standard patients are introduced into the laboratory to make students proficient in physical examination and standardization operation (***Fig. 1B*** and ***1C***). High-fidelity simulators can be used for debriefing and feedback after performance as well as advanced procedures[7]. In the surgical aspect, the laboratory prepares many modules used for suturing and knotting, and asepsis practice for medicos (***Fig. 1D*** and ***1E***). Students then can become familiar with the aseptic principles before they step into the operating theatre. In obstetrics and gynecology, there are vaginal examination models, culdocentesis models, and laboring models, which students can practice on after they have acquired theoretical knowledge (***Fig. 1F***). With the instruments and models, students may practice standardized operation repeatedly. Thus, they will master the normalized procedure and become more and more skillful. After applying simulation-based education into practice, students have more opportunities to be trained in such procedures as puncture, suture, and physical examination. Students also have better scores than before as a result of such training. Medical simulation education has some characteristics which traditional medical education can never catch up with, such as reproducibility, controllability, safety, flexibility and rationality. This new educational mode favors the progress of medicos' clinical skills and can avoid some medical dispute.

Standardized patient (SP) is also called simulated patient. In health care, SP is an individual who is trained to act as a real patient in order to simulate a set of symptoms or problems. Simulated patients have been successfully used in medical education, evaluation, and research. We have invested heavily in the project, which aims at training a group of standardized patients. Now these SPs have been applied in clinical skill training and this mode has achieved certain progress. SPs are able to provide cases that are needed for students and students can interact with them, proceeding to obtain a history, direct the physical examination, identify changes in patient condition, recommends a treatment and monitor for adequate response to therapy[8],[9]. SPs are likely to be more reliable, and may tolerate more students than real patients. They can create vivid clinical scenarios which allow direct comparison of the students' clinical skills. They provide a standardized experience for all students in a safe environment[10]. Students can proactively participate in the clinical teaching. Besides, medicos can do practice at any time they want and learn about situations they may not be able to manage alone in a real clinical setting. Clinical skills involve problem solving skills and the ability to work as a member and to communicate effectively[11]. Dealing with SPs helps strengthen medicos' overall qualities, including their communication skills and clinical thinking abilities.

## CONCLUSION

Medical simulation education creates a convenient, safe and efficient environment for both medical students and physicians. It allows educators to control the environment and ensure that desired learning objectives are met while permitting increased student autonomy without incurring patient safety risks[12]. Besides, it reduces the need for supervision of medical students by physician faculty during clinical encounters. Medical simulation education helps to improve medicos' clinical skills to a large degree and is the inevitable trend in modern medical education. Medical simulation education is evolving through continuing collaboration across different clinical fields and settings[13] and it is an invaluable approach to teach clinical skills and competency required for health care. Overall, medical simulation education has infused new life and energy into medical education.
